# Higher-rate relativistic quantum key distribution

**DOI:** 10.1038/s41598-021-02739-5

**Published:** 2021-12-07

**Authors:** Georgi Bebrov

**Affiliations:** Telecommunications Department, Technical University of Varna, Varna, 9010 Bulgaria

**Keywords:** Quantum information, Single photons and quantum effects

## Abstract

One of the major problems in the field of quantum key distribution (QKD) is the low key rates at which the systems operate. The reasons for this are the processes used to ensure the key distribution itself: *sifting*, *parameter estimation*, *key reconciliation*, and *privacy amplification*. So, this reduction in the rate of communication is inherent to all existing quantum key distribution schemes. This paper is concerned with proposing a solution to mitigate the rate reduction of the so-called relativistic QKD. To mitigate the reduction, we introduce a modified relativistic QKD protocol, which is based on Mach–Zehnder interferometer being used as a *probabilistic basis selection system* (basis misalignment occurs between the parties in approximately half of the transferred qubits). The interferometric scheme allows the participating parties to correlate the mutual unbiased bases (MUBs) chosen by them. In this regard, a qubit could be used to transfer more than one bit of information. To be precise, by implementing the proposed interferometric scheme into a relativistic QKD protocol, a qubit is able to transfer two bits of information. This results in achieving a protocol, which is characterized with a greater rate of communication, two times greater than the usual rate. The modified protocol is proven to be secure against intercept-resend and collective attacks.

## Introduction

The provision of confidentiality is one of the utmost tasks in the field of communication networks. A solution to this task is the usage of the so-called quantum key distribution (QKD). It is a process of information-theoretically secure key establishment between two or more parties, which relies on the laws of quantum mechanics. Classical approaches being used to perform such a process are those introduced in Refs.^1−6^. There exist another ways of performing quantum key distribution (or quantum key establishment). They are known as quantum secure communication (QSC) protocols^7–21^. These protocols are divided into two branches: quantum secure direct communication (QSDC)^7–12^ and deterministic secure quantum communication (DSQC)^13–21^. They differ in the way of transferring messages over the communication channel. In QSDC, the message (key) is transferred only by using a quantum channel. In DSQC, an auxiliary classical information is required for reading out a message encoded in a quantum system. The quantum key distribution is a technology required by a lot of communication systems using sensitive data—such as telemedicine systems, controlling systems (e.g., the communication system of Smart Grid), banking systems and etc. We could point out that the confidentiality is also relevant for an artificial intelligence (AI) unit, if the latter plays a vital role in the above list of systems.

QKD has been developed throughout the years in order for its security to be improved^22–24^. The security is improved by preventing practical loopholes. This is done by introducing the measurement-device-independent or just device-independent schemes^24–40^. Another model, which constrains the eavesdropper, is the so-called relativistic quantum key distribution^41–43^. It is based on putting relativistic limitations to an eavesdropper: “... They allow to force Eve make decisions about her actions before she can actually measure the state in the line, thus breaking her only winning strategy due to causality”^42^. In other words, the relativistic model does not give Eve any chance to launch an attack in due time so that she cannot gather information about the transferred data in an unhindered manner.

A quantum key distribution is a process consisted of the following procedures: (i) quantum encoding, (ii) transfer of quantum systems, (iv) quantum decoding (including sifting), (v) parameter estimation, (vi) error correction^44^, (vii) privacy amplification^45^. The problem of quantum key distribution is the low key rates that the distinct models are characterized with. This drawback is due to implementing the procedures of sifting, parameter estimation, error correction, and privacy amplification. A solution to this problem is the optimization of the procedures incorporated into the QKD (e.g., using high-dimensional quantum systems during the transfer).

This paper is concerned with introducing a new approach of implementing a relativistic protocol, which is characterized with higher rate in establishing sifted keys. The higher rate comes from using more than one mutually unbiased bases (MUBs) for transferring more than one bit per qubit—a more optimal transfer of data by qubits. This is achieved via an interferometric scheme^4^, which could be leveraged in a way that two MUBs are distinguished by introducing certain phase shift at each of its arms (at each arm of the interferometer).

## Methods

In this section, we present an interferometric scheme (practical scheme) that is used for a basis of the key distribution proposed later on. The scheme consists of a Mach–Zehnder interferometer (MZI) involving phase shifts at its arms^4,41^. The following notation is used hereafter: $\left| {z+}\right\rangle$ and $\left| {z-}\right\rangle$ represent the eigenstates of the *Z* operator (rectilinear polarization operator or $\sigma _z$ operator); $\left| {x+}\right\rangle$ and $\left| {x-}\right\rangle$ represent the eigenstates of the *X* operator (diagonal polarization operator or $\sigma _x$ operator). As is known, the eigenstates of a given operator form a basis: the states $\left| {z+}\right\rangle$ and $\left| {z-}\right\rangle$ form the so-called *Z* basis, whereas the states $\left| {x+}\right\rangle$ and $\left| {x-}\right\rangle$ form the so-called *X* basis. We should note that the two bases are interconnected through the following relations

1 $$\begin{aligned} \left| {z+}\right\rangle = \frac{\left| {x+}\right\rangle + \left| {x-}\right\rangle }{\sqrt{2}}, \; \left| {z-}\right\rangle = \frac{\left| {x+}\right\rangle - \left| {x-}\right\rangle }{\sqrt{2}}. \end{aligned}$$

In terms of polarization, the states $\left| {z+}\right\rangle$ and $\left| {z-}\right\rangle$ respectively correspond to vertical and horizontal polarizations, whereas the states $\left| {x+}\right\rangle$ and $\left| {x-}\right\rangle$ respectively correspond to diagonal and off-diagonal polarizations.

**Figure 1:**
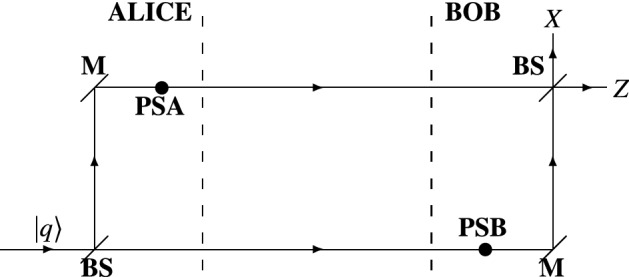
Mach–Zehnder interferometer used to transfer two-bit message via a single qubit system. *PSA* phase shift possessed by Alice, *PSB* phase shift possessed by Bob, $\left| {q}\right\rangle$ input qubit state, *BS* beam splitter, *M* mirror, *Z* *Z*-basis measurement system, *X* *X*-basis measurement system.

Now we consider the Mach–Zehnder interferometer depicted in Fig. 1. Compared to the interferometer of Ref.^46^, the present scheme involves phase shifts (**PSA** and **PSB**), one at each arm of the interferometer^4,41^. The phase shifts are used to control the output at which a given input system $\left| {q}\right\rangle$ goes through. Note that each phase shift can take only two values (90-deg or 270-deg). The current interferometric scheme resembles that of Ref.^4^. As pointed out and thoroughly explained in Refs.^41,46^, when **PSA** = **PSB** (or there are no phase shifts in the scheme) the input qubit $\left| {q}\right\rangle$ goes through the upper output (*X*-basis output of the scheme, see Fig. 1). Otherwise (when **PSA** $\ne$ **PSB**), the qubit goes through the lower output (*Z*-basis output). We should note that the global phase of the output qubit $\left| {q}\right\rangle$ is neglected, because it does not play a role in a measurement procedure after all. A given measurement system (*X*-basis or *Z*-basis system) consists of polarization beam splitter and two detectors, which distinguish the orthogonal states of a given basis.

By means of the presented interferometric scheme, Alice communicates two-bit messages (key words) per qubit to Bob: four qubit states of $\left| {q}\right\rangle$ (eigenstates of *X* basis and eigenstates of *Z* basis) are used to convey information. More details on the process of transferring messages (key bits) are given in the next section.

## Results

### Key distribution scheme

To begin the key distribution, Alice prepares a random sequence of qubits $\left| {q}\right\rangle _i$, using states $\left| {z+}\right\rangle$, $\left| {z-}\right\rangle$, $\left| {x+}\right\rangle$, and $\left| {x-}\right\rangle$ to represent the messages 00, 01, 10, and 11, respectively. She then puts the systems through the Mach–Zehnder interferometer (MZI) presented in Fig. 1. Alice performs a phase shift (operator) **PSA** on each quantum system (qubit) $\left| {q}\right\rangle$: the phase shift is chosen at random regardless of the $\left| {q}\right\rangle _i$ state. Next the quantum systems travel to the Bob’s side of the interferometer. He performs a phase shift **PSB** of either 90- or 270-deg in a random manner. Based on the phase shifts **PSA** and **PSB**, the quantum systems take one of the two possible outputs of the MZI: if **PSA** = **PSB** a qubit goes through the *X*-basis output, otherwise a qubit goes through the *Z*-basis output. Bob records the measurement result of each qubit (Bob determines the detector, which registers a click, when a given qubit is measured). Next Bob announces over an authenticated public classical channel his phase shifts **PSB**$_i$. The reason why Bob chooses at random his phase shifts is the fact that he will subsequently announce them over a public classical channel. Since the choice of phase shifts by Bob are random and independent of Alice, the public announcement does not leak information to an eavesdropper, because no relevant information is revealed about the bases and states of the transferred/received qubits. Information would leak only if, for instance, Bob announces the phase shifts together with his measurement results.

Based on **PSB**$_i$, **PSA**$_i$, and the bases of the qubits prepared by her, Alice decides which quantum systems should be discarded due to basis misalignment (a *sifting* process is conducted). The decision is made in terms of the following requirement

2 $$\begin{aligned} (\mathbf{PSA} _i\oplus \mathbf{PSB} _i) \oplus (\mathbf{BASIS} _i) = 1. \end{aligned}$$

Here **BASIS**$_i$ stands for a bit, which reflects the basis of a qubit $\left| {q}\right\rangle _i$: qubit in a *Z* basis $\rightarrow$ **BASIS**$_i$ = 0; qubit in a *X* basis $\rightarrow$ **BASIS**$_i$ = 1. In this expression, **PSA**$_i$ and **PSB**$_i$ takes the following binary values: **PSA**$_i$ = **PSB**$_i$ = 0 when 90-deg phase shift is performed; **PSA**$_i$ = **PSB**$_i$ = 1 when 270-deg phase shift is performed. If the above requirement is met, Alice and Bob do not discard the measurement result of a given qubit system $\left| {q}\right\rangle _i$. In this case, 50% of the initial qubits are discarded (as in the case of standard, original QKD). However, the remaining qubits (50%) carry twice the amount of bits compared to the original scheme. This is so, because each qubit could be in either of four qubit states: $\left| {z+}\right\rangle$, $\left| {z-}\right\rangle$, $\left| {x+}\right\rangle$, $\left| {x-}\right\rangle$.

In order to show that the above sifting process does not leak information to the eavesdropper, a mathematical analysis is presented. The analysis examines the amount of information that an eavesdropper gathers when certain information (random variable) is publicly announced. We consider two cases in the analysis: (1) a random variable, which is not related to the bases of the transferred qubits, is publicly announced; and (2) a random variable, which is related to the bases of the transferred qubits, is publicly announced. Note that case (1) reflects the process of Bob’s announcing phase shifts. In order to quantify the amount of information, which Eve obtains when public announcement is conducted, we utilize the concept of mutual information^47^

3 $$\begin{aligned} I(V;W) = H(V) - H(V|W). \end{aligned}$$

#### Case (1)

In this expression, *V* is a variable playing the role of the bases in which the qubits are prepared and *W* is a variable playing the role of the phase shifts announced by Bob (*V*: *v* $\in$ {0,1}: 0 $\rightarrow$ *Z* basis, 1 $\rightarrow$ *X* basis; *W*: *w* $\in$ {0,1}: 0 $\rightarrow$ **PSB** = 90-deg, 1 $\rightarrow$ **PSB** = 270-deg). We know from above that both *V* and *W* are chosen at random so that *H*(*V*) = *H*(*W*) = 1 (this implies $Pr(V=v)$ = $Pr(W=w)$ = 0.5). *H*(*V*) = *H*(*W*) = 1 also shows that the bases of the qubits and the phase shifts are truly random from Eve’s standpoint. We now need to determine *H*(*V*|*W*). It is given by

4 $$\begin{aligned} H(V|W) = \sum _{w=0}^1 Pr(W=w)H(V|W=w), \end{aligned}$$

where

5 $$\begin{aligned} H(V|W=w) = - \sum _{v=0}^1 Pr(V=v|W=w)log_2Pr(V=v|W=w), \end{aligned}$$

As described above, the variables *V* and *W* are independent (*Note*: They are chosen from two different persons and for the sake of two different processes (preparation of qubits and phase shifting)). This implies that $Pr(V=v|W=w)$ = $Pr(V=v)$. Therefore, Eq. (5) transforms into

6 $$\begin{aligned} H(V|W=w) = - \sum _{v=0}^1 Pr(V=v)log_2Pr(V=v) \end{aligned}$$

and has the value of

7 $$\begin{aligned} H(V|W=w) = - \sum _{v=0}^1 0.5log_2 0.5 = 1. \end{aligned}$$

This leads to

8 $$\begin{aligned} H(V|W) = \sum _{w=0}^1 Pr(W=w)H(V|W=w) = \sum _{w=0}^1 0.5\times 1 = 1. \end{aligned}$$

Taking into account the result of the last expression and that *H*(*V*) is equal to unity (*H*(*V*) = 1), we obtain for the mutual information:

9 $$\begin{aligned} I(V;W) = H(V) - H(V|W) = 1 - 1 = 0 \; bits, \end{aligned}$$

which means that Eve does not gain information about the bases of the transferred qubits given that Bob announces his phase shifts.

#### Case (2)

We consider the case when a given random variable *U* (*U*: *u* $\in$ {0,1}), which is related to the bases of the transferred qubits (related to variable *V*, see the previous case for reference), is publicly announced. Suppose that *V* and *U* are completely correlated: if *V*: *v* = 0, then for sure *U*: *u* = 0. As with the previous scenario, $Pr(V=v)$ = 0.5. Also, from the correlation just mentioned, we infer that $Pr(U=u)$ = 0.5. The complete correlation between *V* and *U* leads to the result $Pr(V=v|U=u)$ = 1. This comes from the fact that we always have *V* = *v* (e.g., *v* = 0) whenever *U* = *u* (when *u* = 0). Taking this into account, we obtain for $H(V|U=u)$:

10 $$\begin{aligned} H(V|U=u) = - \sum _{v=0}^1 Pr(V=v|U=u)log_2Pr(V=v|U=u) = 0. \end{aligned}$$

This result, in turn, leads to

11 $$\begin{aligned} H(V|U) = \sum _{u=0}^1 Pr(U=u)H(V|U=u) = \sum _{w=0}^1 0.5\times 0 = 0. \end{aligned}$$

Then, for the mutual information *I*(*V*; *U*) we get

12 $$\begin{aligned} I(V;U) = H(V) - H(V|U) = 1 - 0 = 1 \; bit. \end{aligned}$$

We can interpret *I*(*V*; *U*) = 1 as that the eavesdropper is aware of the bases in which the transferred qubits $\left| {q}\right\rangle _i$ are prepared, regardless of the information announced by Bob about his phase shifts provided that *U* is an additionally announced information. The current scenario demonstrates that the information about the basis of a transferred qubit state is solely contained in the correlation between *U* and *V*. Therefore, a publicly announced random variable, which is not related (or correlated) to *V* (bases of the qubits), does not leak information to the eavesdropper.

In this way, by the above analysis, we show that the phase shift **PSB** of Bob, being an independent random variable, does not give information to third parties about the bases of transferred qubit states (correspondingly, a part of a message transferred by each qubit). Therefore, knowing only the phase shifts of Bob, Eve cannot determine whether **PSA**$_i$ = **PSB**$_i$ or **PSA**$_i$ $\ne$ **PSB**$_i$, i.e., whether $\left| {q}\right\rangle _i$ is prepared in *X* basis or $\left| {q}\right\rangle _i$ is prepared in *Z* basis. Being unaware of the basis of a qubit, Eve is also ignorant of the state of this qubit—this is a result of the fact that she cannot perform an appropriate measurement on the qubit in order to determine its actual state.

In the next lines, for the sake of clarity, we work out an example of transferring messages via several quantum systems by means of the interferometric scheme proposed above. The example demonstrates how a sifted key is obtained in a quantum key distribution using the scheme of concern. Suppose Alice intends to send to Bob the following sequence of quantum states,

**Table:** 

$\left| {q}\right\rangle _0$	$\left| {q}\right\rangle _1$	$\left| {q}\right\rangle _2$	$\left| {q}\right\rangle _3$	$\left| {q}\right\rangle _4$
$\left| {x-}\right\rangle$	$\left| {z-}\right\rangle$	$\left| {z+}\right\rangle$	$\left| {z-}\right\rangle$	$\left| {x+}\right\rangle$

which correspond to the following two-bit symbols.

$$\begin{aligned} 11\, 01 \, 00 \, 01 \, 10. \end{aligned}$$

Note that not all of the two-bit symbols will be properly decoded by Bob. In other words, some of the symbols will be discarded. The two-bit symbols, correspondingly the qubit states, are prepared by way of a random choice. Next Alice sends the states through the interferometric scheme of Fig. 1. Alice at random chooses to perform the following **PSA** phase shifts:

**Table:** 

PSA$_0$	PSA$_1$	PSA$_2$	PSA$_3$	PSA$_4$
90-deg	270-deg	270-deg	90-deg	270-deg

Suppose Bob at random chooses the following **PSB** phase shifts, which are applied at the lower arm of the interferometer:

**Table:** 

PSB$_0$	PSB$_1$	PSB$_2$	PSB$_3$	PSB$_4$
90-deg	270-deg	90-deg	90-deg	270-deg

According to the description above, if **PSA**$_i$ = **PSB**$_i$ a qubit goes through the upper output, otherwise it goes through the lower output. In this regard, we have in the example that $\left| {q}\right\rangle _0$, $\left| {q}\right\rangle _1$, $\left| {q}\right\rangle _3$ and $\left| {q}\right\rangle _4$ leave the interferometer through the upper output (*X*-basis measurement system), whereas $\left| {q}\right\rangle _2$ leaves the interferometer through the lower output (*Z*-basis measurement system). This implies that $\left| {q}\right\rangle _0$, $\left| {q}\right\rangle _2$, and $\left| {q}\right\rangle _4$ will be measured in appropriate bases, while the other quantum systems will be measured in inappropriate bases. Bob publicly announces the following binary string, whose elements correspond to the **PSB** phase shifts he chose (**PSB**$_i$ = 90-deg $\rightarrow$ 0; **PSB**$_i$ = 270-deg $\rightarrow$ 1),

$$\begin{aligned} 0\, 1\, 0\, 0\, 1. \end{aligned}$$

Using this string (corresponding to the phase shifts of Bob), her phase shifts (**PSA**$_i$), and the bases of her initial states, Alice determines if each qubit $\left| {q}\right\rangle _i$ (detected by Bob) satisfies Eq. (2). We should note that the string **BASIS** is comprised of the following bits, taking into account the states of $\left| {q}\right\rangle _i$ prepared by Alice,

**Table:** 

BASIS$_0$	BASIS$_1$	BASIS$_2$	BASIS$_3$	BASIS$_4$
1	0	0	0	1

As mentioned above, an element of the string **BASIS** (**BASIS**$_i$) has a value of 0 if a qubit $\left| {q}\right\rangle _i$ is prepared in *Z* basis and a value of 1 if a qubit is prepared in *X* basis. This is reflected in the above table. Now we present a table that depicts a binary string **D**, whose elements **D**$_i$ show if each qubit $\left| {q}\right\rangle _i$ satisfies Eq. (2) (**D**$_i$ = 1 $\rightarrow$ $\left| {q}\right\rangle _i$ satisfies Eq. (2); **D**$_i$ = 0 $\rightarrow$ $\left| {q}\right\rangle _i$ does not satisfy Eq. (2):

**Table:** 

D$_0$	D$_1$	D$_2$	D$_3$	D$_4$
1	0	1	0	1

In order to be as clear as possible in terms of this process (the process is actually a *sifting* procedure), we in detail determine the values of **D**$_i$. Based on the values of **D**$_i$, Alice and Bob discard (sift) certain quantum systems (**D**$_i$ = 1 $\rightarrow$ $\left| {q}\right\rangle _i$ remains; **D**$_i$ = 0 $\rightarrow$ $\left| {q}\right\rangle _i$ is discarded). As is known from above, **PSA**$_0$ = 0 (corresponding to 90-deg), **PSB**$_0$ = 0, and **BASIS**$_0$ = 1. Substituting the values of these quantities in Eq. (2), we obtain

$$\begin{aligned} (\mathbf{PSA} _0\oplus \mathbf{PSB} _0) \oplus (\mathbf{BASIS} _0) = (0 \oplus 0) \oplus (1) = 1. \end{aligned}$$

This implies that **D**$_0$ = 1, that is, the first received qubit satisfies Eq. (2). Taking into account the remaining values (*i* = 1,2,3,4) of **PSA**$_i$, **PSB**$_i$, and **BASIS**$_i$, we have for the remaining elements **D**$_i$:

$$\begin{aligned}&(\mathbf{PSA} _1\oplus \mathbf{PSB} _1) \oplus (\mathbf{BASIS} _1) = (1 \oplus 1) \oplus (0) = 0. \\&(\mathbf{PSA} _2\oplus \mathbf{PSB} _2) \oplus (\mathbf{BASIS} _2) = (1 \oplus 0) \oplus (0) = 1. \\&(\mathbf{PSA} _3\oplus \mathbf{PSB} _3) \oplus (\mathbf{BASIS} _3) = (0 \oplus 0) \oplus (0) = 0. \\&(\mathbf{PSA} _4\oplus \mathbf{PSB} _4) \oplus (\mathbf{BASIS} _4) = (1 \oplus 1) \oplus (1) = 1. \end{aligned}$$

Alice publicly announces the string **D**. In this way, Alice informs Bob about the quantum systems that should be sifted (discarded). As a result, only the two-bit messages transferred by $\left| {q}\right\rangle _0$, $\left| {q}\right\rangle _2$, and $\left| {q}\right\rangle _4$ remain: $\left| {q}\right\rangle _0$ $\rightarrow$ 11, $\left| {q}\right\rangle _2$ $\rightarrow$ 00, $\left| {q}\right\rangle _4$ $\rightarrow$ 10, see the beginning of the example for reference. These two-bit messages form the so-called sifted key.

### Relativistic QKD protocol

**Figure 2:**
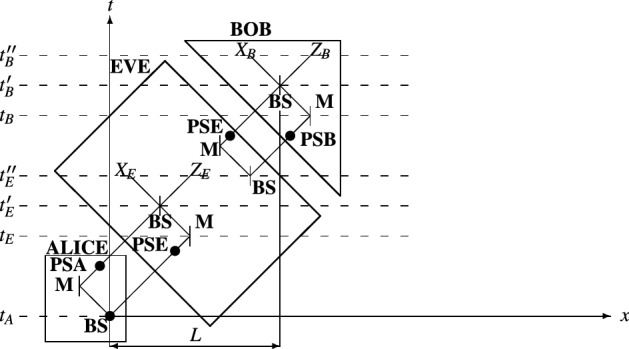
Space-time diagram for the proposed relativistic QKD protocol. *PSA* phase shift of Alice; *PSB* phase shift of Bob; *PSE* phase shift of Eve; *BS* beam splitter; *M* mirror; *Z* *Z*-basis measurement system; *X* *X*-basis measurement system.

In this section, we introduce a relativistic quantum key distribution protocol, which could be regarded as a modified version of the scheme in Ref.^42^ or that of Ref.^43^. The proposed protocol requires one-photon source and completely synchronized clocks being at the disposal of both sender and recipient (Alice and Bob). A schematic description of the novel relativistic protocol is shown in Fig. 2. The distance *L* between the beam splitter of Alice and the beam splitter of Bob is preliminary, publicly known. The proposed protocol makes use of the interferometric scheme introduced above. The protocol is characterized with the following steps:

1. At time $t_A$ (*t* = 0) Alice prepares single-photon qubit states $\left| {q}\right\rangle _i$ ($\left| {q}\right\rangle _i$ $\in$ {$\left| {z+}\right\rangle$,$\left| {z-}\right\rangle$,$\left| {x+}\right\rangle$,$\left| {x-}\right\rangle$}) and puts the system into her beam splitter (into the interferometric scheme). As in Refs.^42,43^, the instant $t_A$ is regarded as the beginning of the protocol. After passing the beam splitter, the quantum system jumps over into a superpositional state of the interferometer. The superposition is a combination of the states in the lower and upper arms of the interferometer: ($\left| {q}\right\rangle ^l_i$ + $e^{i\frac{\pi }{2}}\left| {q}\right\rangle ^u_i$)/$\sqrt{2}$, where the phase of the second state reflects the 90-deg change in the path direction (a direction towards the mirror **M**)^46^.
2. Alice at random performs a phase shift **PSA** of either 90-deg or 270-deg, see section “Key distribution scheme” for a reference.
3. The qubit $\left| {q}\right\rangle _i$ travels to Bob’s side. Bob performs a phase shift **PSB**$_i$ of either 90-deg or 270-deg in a random manner on each qubit $\left| {q}\right\rangle _i$.
4. At time $t_B'$ the qubit $\left| {q}\right\rangle ^u_i$ of the upper path interferes with the qubit $\left| {q}\right\rangle ^l_i$ of the lower path. Based on the values of **PSA** and **PSB**, $\left| {q}\right\rangle _i$ leaves the interferometer either through the *X*-basis or *Z*-basis output, as described above.
5. At time $t_B''$ Bob measures the state $\left| {q}\right\rangle _i$ via either *X*-basis or *Z*-basis measurement system. He records the measurement outcomes. Note that Bob discards the measurements of qubits, which take place latter than $t_B''$. He regards the retarded qubits as counterfied (or eavesdropped).
6. Next Bob announces the phase shifts **PSB**$_i$ performed on each $\left| {q}\right\rangle _i$ and the qubits being retarded.
7. As described above, Alice decides which non-retarded qubits $\left| {q}\right\rangle _i$ are further discarded. The decisions are taken according to Eq. (2). She informs Bob about her decisions.
8. Parameter estimation is carried out by Alice and Bob. They sacrifice part of the quantum systems in order to evaluate the quantum bit error rate of the communication channel. If the error rate exceeds a preliminary determined threshold, Alice and Bob terminate the current session and start over the protocol. Otherwise, they proceed forward.
9. Alice and Bob conduct the so-called error reconciliation process^44^.
10. Alice and Bob conduct the so-called privacy amplification process^45^.

## Discussion

In this section, we discuss the security and performance of the protocol proposed in the previous lines. Also, we propose a practical implementation of the proposed protocol, which makes use of weak coherent pulses.

### Security analysis

We analyze the security of the proposed protocol in terms of several attacks^15,43^: (i) intercept-resend attack, (ii) intercept-resend attack with preliminary prepared state, (iii) collective attack.

(i) Because the initial state $\left| {q}\right\rangle$ is spread over the space-time ($\left| {q}\right\rangle$ is divided into $\left| {q}\right\rangle _l$ and $\left| {q}\right\rangle _u$), the eavesdropper needs to have an access to both $\left| {q}\right\rangle _l$ and $\left| {q}\right\rangle _u$ in order to acquire the actual state of the qubit $\left| {q}\right\rangle$. For this purpose, Eve is required to utilize a receiving side of the interferometric scheme introduced above, as shown in Fig. 2, in order to recombine $\left| {q}\right\rangle _l$ and $\left| {q}\right\rangle _u$ into $\left| {q}\right\rangle$. By doing so, Eve disturbs the initial path of $\left| {q}\right\rangle _l$. Also, performing a measurement on $\left| {q}\right\rangle$ leads to disturbance of the space-time path of $\left| {q}\right\rangle _u$, too. Given that a measurement is conducted, the $\left| {q}\right\rangle _u$ resent by Eve will not reach Bob’s beam splitter at a correct time. This results in erroneous interference at time $t_B'$. This holds for the $\left| {q}\right\rangle _l$ as well. It is possible for $\left| {q}\right\rangle _l$ to reach Bob’s beam splitter at time $t_B'$ (if and only if $Z_E$ measurement is conducted), but this partial state will interfere with nothing—it will be the only partial state that will reach Bob’s beam splitter at time $t_B'$. In this case, the beam splitter outputs a qubit in an erroneous path ($X_B$ or $Z_B$) with probability of 1/2, as described in Ref.^46^. In the case of $X_E$ measurement, both $\left| {q}\right\rangle _l$ and $\left| {q}\right\rangle _u$ resent by Eve cannot reach Bob’s beam splitter at the correct time. This results in conducting no measurement at Bob’s side. Bob interprets such an absence of measurement as an act of eavesdropping. Therefore, Eve disturbs the quantum system $\left| {q}\right\rangle$ by performing measurement and the disturbance is completely detectable by Alice and Bob.
(ii) In this attack, the eavesdropper prepares a qubit $\left| {q}\right\rangle ^e$ ($\left| {q}\right\rangle ^e$ $\in$ {$\left| {z+}\right\rangle$,$\left| {z-}\right\rangle$,$\left| {x+}\right\rangle$,$\left| {x-}\right\rangle$}), whose state is randomly chosen. Eve also intercepts the Alice’s qubit $\left| {q}\right\rangle$ by using a receiving side of the interferometric scheme. On intercepting the qubit $\left| {q}\right\rangle$, Eve sends $\left| {q}\right\rangle ^e$ to Bob via a transmitting side of the interferometric scheme (she uses the same setup as Alice). In this case, a proper interference always occurs at time $t_B'$. However, the interference could be that of an incorrect state of $\left| {q}\right\rangle ^e$, because Eve’s qubit has no correlation to the Alice’s qubit. This results in disturbing the quantum channel between Alice and Bob. In other words, Eve causes error in the initial state of Alice’s qubit $\left| {q}\right\rangle$. The probability of error is 3/4—in only 1/4 of the cases Eve correctly guesses the initial state of Alice’s qubit. In this way, Alice and Bob can detect the presence of Eve during the parameter estimation procedure (evaluating the error rate of the channel).
(iii) In the collective attack, the eavesdropper appends an ancilla $\left| {a}\right\rangle$ to the Alice’s qubit $\left| {q}\right\rangle$ in order to gain information about the state of $\left| {q}\right\rangle$ in an unhindered manner. To do so, Eve performs a unitary operator on the system $\left| {q}\right\rangle \left| {a}\right\rangle$ so that the ancilla gets changed according to the state of $\left| {q}\right\rangle$. As described in Ref.^15^, such a unitary operation is the so-called CNOT gate, which flips the ancilla state if $\left| {q}\right\rangle$ is in $\left| {z-}\right\rangle$ state. However, the CNOT gate is appropriate only in the case when $\left| {q}\right\rangle$ is prepared in either $\left| {z+}\right\rangle$ or $\left| {z-}\right\rangle$ (in *Z* basis). In the case when $\left| {q}\right\rangle$ is prepared in the *X* basis, the CNOT gate sets the ancilla in a superposition ($\left| {z+}\right\rangle$ + $\left| {z-}\right\rangle$)/$\sqrt{2}$, which gives information about $\left| {q}\right\rangle$ if and only if the ancilla is measured in the *X* basis. We have the same picture when a CNOT gate in terms of the *X* basis is constructed and used during this attack. The problem of this kind of attack is that the eavesdropper needs to correctly guess the basis in which the state of $\left| {q}\right\rangle$ is prepared. As mentioned above, the random guess leads to presenting an error in the state of $\left| {q}\right\rangle$ with certain probability. During the parameter estimation process (see section Relativistic QKD Protocol), Alice and Bob can detect the errors induced by Eve when the collective attack being launched. Therefore, the presence of an eavesdropper is detectable during this attack, too.

**Figure 3:**
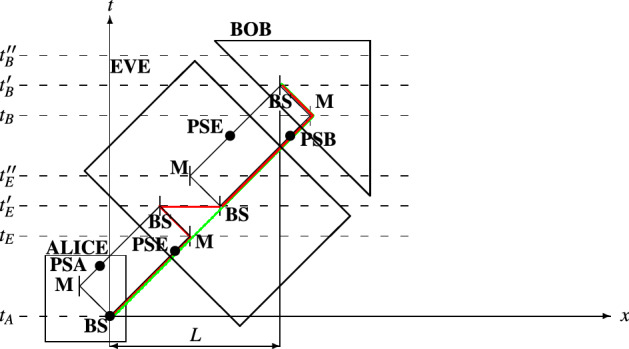
Comparison between two space-time paths: (red)—a lower path of an interfering qubit, which is intercepted; (green)—a lower path of an interfering qubit, which is not intercepted.

In the next lines, we present in more details the relativistic security of the proposed protocol against the attacks concerned above, which are called *intercepting attacks* hereafter. For the sake of the analysis, Fig. 3 is presented. We assume that the measurement systems are assumed to be located maximally close to the corresponding beam splitters. That is why we omit displaying the measurement systems in Fig. 3.

As is known, the velocity of light *c* is a constant regardless of one’s frame of reference^48–50^. Taking a look at Fig. 1, this implies that a photon transmitted from Alice’s beam splitter (at $t_A$) should reach Bob’s beam splitter at time $T_l$ = $t_B'$ − $t_A$, crossing a distance of $L_l$ = *c*($t_B$ − $t_A$) + *c*($t_B'$ − $t_B$) provided that an eavesdropper does not intercept the particle. This holds for a photon travelling through the lower arm of the interferometric scheme. In the case when the photon takes the path of the upper arm, it arrived at Bob’s beam splitter at time $T_u$ = $t_B'$ − $t_A$ and traverse a distance of $L_u$ = *c*($t_A'$ − $t_A$) + *c*($t_B'$ − $t_A'$). As could be readily seen, both upper and lower paths of the photon are characterized with the same space-time features (distance *L* = $L_l$ = $L_u$ and time interval *T* = $T_l$ = $T_u$).

If an eavesdropper intercepts a photon, as shown in Fig. 1, she slightly alters particle’s path. Intercepting a particle, Eve causes a change in trajectory, which moves a lower path particle into the upper path. If Eve tries to maintain the relativistic characteristics of the scheme, she somehow needs to return a measured particle (for instance, particle measured in the *Z* basis) back at the lower path at time $t_E''$. Another solution to this problem is to generate (or prepare in a certain state) a particle at $t_E''$ after the measurement of the original particle. According to relativity, the two cases are impossible. This is pictorially illustrated in Fig. 3. In the figure, two paths are presented: (green)—this is the natural path of an interfering qubit, which travel along the lower path of the interferometric scheme; (red)—this is the path taken by an interfering qubit when Eve launches intercepting attack. First, it is impossible for Eve to move a particle from the measurement systems to the lower path of the Alice-Bob interferometer. As can be seen from Fig. 3, the particle needs to travel at speed exceeding *c* (the slope of the displacement (receiving **BS** to transmitting **BS** of Eve) is of space-like type). Second, it is impossible for Eve to prepare a particle at one location in a state conditioned on a measurement taking part at another location, given that the two events (preparation and measurement) occur at the same time. In this case, the information obtained from the measurement, which is necessary for preparing a state at another location) should travel at a speed exceeding that of light. As is known, the latter is impossible according to the relativistic principles. The above analysis could be mathematically verified as follows. Intercepting the interferometric communication between Alice and Bob, Eve introduces longer space-time path of the interfering qubits. For instance, as shown in Fig. 3, the distance of the lower interferometric path $L_l$ gets longer when interception occurs. In this way, Bob receives retarded detection counts, which reveals the presence of Eve. In order to show that $L_l$ (displayed in green color in Fig. 3) gets longer, we calculate its counterpart $L_l'$ (displayed in red color), which is the path involving the interception. The distance $L_l'$ is the following

13 $$\begin{aligned} L_l' = (t_E - t_A)c + (t_E' - t_E)c + (t_E' - t_E')c + (t_B - t_E')c + (t_B' - t_B)c. \end{aligned}$$

From Fig. 3, it is evident that ($t_E'$ − $t_E$)*c* = ($t_B'$ − $t_B$)*c*. In the last equation, there is a term of singularity (($t_E'$ − $t_E'$)*c*), which we omit in the comparison between $L_l$ and $L_l'$. The term of singularity appears to be a manifestation of instantaneous displacement of an object from one point to another, which is a concept prohibited by the theory of relativity. This verifies the analysis in the above lines about the impossibility of Eve to prepare a duplicate qubit of the intercepted (measured) one. We now find the difference between $L_l$ and $L_l'$:

14 $$\begin{aligned} L_l' - L_l = [(t_E - t_A)c + (t_E' - t_E)c + (t_B - t_E')c + (t_B' - t_B)c] - [(t_B - t_A)c + (t_B' - t_B)c] = (t_E' - t_E)c. \end{aligned}$$

Note that ($t_B$ − $t_A$)*c* = ($t_E$ − $t_A$)*c* + ($t_E'$ − $t_E$)*c* + ($t_B$ − $t_E'$)*c*, see Fig. 3 for reference. The above result shows that the interception path $L_l'$ is longer than the natural path $L_l$ of interferometric scheme shared between Alice and Bob. In this way, we mathematically prove that Bob will always received a retarded qubit (detector count) if an intercepting attack is launched.

The only solution for Eve is to preliminary prepare at random a state (particle) and at time $t_E''$ put it through the interferometer, which she shares with Bob. However, being unaware of the particle state transferred by Alice, Eve could prepare her particle in a wrong state (also choose a wrong phase shift **PSE** at Eve-Bob interferometer) and thus cause an erroneous detection, which reveals her presence.

In the previous lines, we show that any action of the eavesdropper is detectable by Alice and Bob in some instant of the quantum key distribution protocol by using either space-time features or uncertainty principle of quantum mechanics or single-particle interference phenomenon. Therefore, we can point out the following features being the tools in discovering the presence of third parties in the course of any intercept-resend attack launched against the relativistic protocol proposed herein:

- *Any disturbance in the original space-time path of a qubit system is detectable*—an interception (measurement), which requires the completion of photon’s interference process, certainly forces the photon to deviate from its space-time path. In this way, the arriving time of the photon at Bob’s side is delayed. By means of this delay, Bob is capable of detecting the presence of an eavesdropper.
- *Any pre-interference measurement is not allowed*—measurement conducted before the receiving beam splitter (second beam splitter of an interferometer, see Fig. 2) causes destruction of the interference phenomenon. The destruction leads to randomness in the output path of the beam splitter. The randomness, in turn, gives rise to errors in the detection step (quantum states are directed towards erroneous detection system).
- *Any attempt of gaining information out of a qubit with the help of ancilla is not allowed*—in this case, the eavesdropper needs to choose a correct polarization basis of the ancilla in order both to gain information and not to disturb the state of the intercepted qubit.

So, it is possible for an eavesdropper to gather information about the sifted key, but at the cost of revealing her presence.

### Performance analysis

The performance of the transfer stage of a protocol is in principle characterized by the data transfer *rate* and *size* of the established sifted key. Note that the *rate* is measured in [*bits*/*qubit*] and the *size* in [*bits*]. In the following lines, we evaluate the value of the *rate* for the proposed relativistic protocol. We also compare the latter to the values of the existing protocols of the same kind (Refs.^42,43^).

The data transfer rate of a quantum key distribution protocol is expressed as

15 $$\begin{aligned} R = \frac{n}{q}, \end{aligned}$$

where *n* is the *size* of the sifted key, whereas *q* is the amount of transferred qubits. In the proposed protocol, the amount of relevant qubits is $\frac{q}{2}$ given that Alice and Bob have destructive (inappropriate) misalignment in their phase shifts in one half of the transferred qubits. By relevant we mean qubits, which take part in establishing the so-called sifted key. Knowing from the above sections that each relevant qubit transfers a two-bit message (two key bits), we obtain for the *size* of the sifted key: *n* = 2($\frac{q}{2}$) = *q*. Taking into account Eq. (15), the *rate* *R* gets the value of

16 $$\begin{aligned} R = \frac{n}{q} = \frac{q}{q} = 1 \; [bit/qubit]. \end{aligned}$$

Note that in defining the rate *R* we neglect the presence of an eavesdropper so that we do not take into account the qubits, whose space-time paths are disturbed.

In order to assess the performance of the proposed protocol, we compare it to its standard counterparts: the schemes introduced in Refs.^42,43^. To do so, we need to determine both rate and sifted-key size of the standard relativistic QKD protocols introduced in Refs.^42,43^. In Refs.^42,43^, Alice and Bob discard one half of the transferred qubits *q* due to choice of uncertain (inappropriate) measurements^43^ or choice of inappropriate phase shifts^42^ by Bob. So, the relevant amount of qubits are $\frac{q}{2}$. Also, according to^42,43^ each qubit transfers one bit of information (one key bit). This results in establishing a sifted key of *size* *n* = 1($\frac{q}{2}$) = $\frac{q}{2}$. In this regard, the *rate* of the standard relativistic protocols is

17 $$\begin{aligned} R' = \frac{n}{q} = \frac{\frac{q}{2}}{q} = \frac{1}{2} = 0.5 \; [bits/qubit]. \end{aligned}$$

To summarize the comparison between the proposed and standard relativistic protocols, we present Table 1 in which the rates and sifted-key sizes of these protocols are collated. In this way, we show that the performance of the proposed protocol exceeds twice the performance of the standard relativistic QKD protocols. In this connection, the proposed protocol could be regarded as an enhanced version of the standard ones.

**Table 1:** Comparison between proposed and standard^42,43^ relativistic QKD protocols in terms of rate and sifted-key size.

Protocol	Rate	Sifted-key size
Ref.^42^	0.5	$\frac{q}{2}$
Ref.^43^	0.5	$\frac{q}{2}$
Proposed protocol	1	*q*

*q* (the amount of transferred qubits) is an arbitrary positive integer, e.g., *q* = 10$^4$.

### Implementation with weak pulses

In section “Relativistic QKD protocol”, we propose a single-photon model for establishing secret cryptographic keys between two parties. We should note that a protocol implementation with weak coherent pulses (WCP) is feasible. A possible WCP implementation of the relativistic protocol is the scheme introduced in Ref.^51^ (named 4 + 2 Protocol), as we bring slight changes into it. We now introduce a WCP scheme for the relativistic protocol proposed in this article, which is an adaptation of the “4 + 2 Protocol” realization.

**Figure 4:**
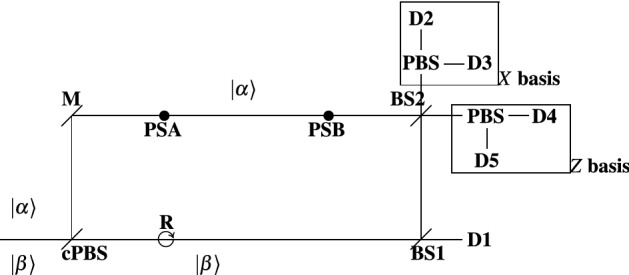
WCP scheme for the protocol proposed in this paper. *PSA* phase shift of Alice; *PSA* phase shift of Bob; $\left| {q}\right\rangle$ input weak coherent state ($\beta$ reference (strong) state; $\alpha$ signal state); *BS* beam splitter; *cPBS* controlled polarization beam splitter; *M* mirror; *R* rotation (through 90-deg); *D* detector.

The WCP implementation of the proposed relativistic protocol is characterized with the schematic illustrated in Fig. 4. Compared to Ref.^51^, the novel scheme involves two phase shifts, two additional detectors, and two additional polarization beam splitters. Also, the polarization beam splitter in the beginning of the interferometer is controllable. This implies that the device is tuned to perform eigenstates discrimination in any polarization basis. In the proposed scheme, **cPBS** is used to discriminate both the eigenstates of the *X* basis (**xPBS**) and the eigenstates of the *Z* basis (**zPBS**). At the input of the interferometric scheme, two orthogonal WCP states are fed: $\left| {\alpha }\right\rangle$ and $\left| {\beta }\right\rangle$. As in Ref.^51^, the state $\left| {\beta }\right\rangle$ is a reference state and $\left| {\alpha }\right\rangle$ is a signal state. Another modification is the fact that the WCP states, which are used in the scheme, could reside in either of the polarization states: {$\left| {V}\right\rangle$ = $\left| {z+}\right\rangle$,$\left| {H}\right\rangle$ = $\left| {z-}\right\rangle$,$\left| {D}\right\rangle$ = $\left| {x+}\right\rangle$,$\left| {A}\right\rangle$ = $\left| {x-}\right\rangle$}. In other words, $\left| {\alpha }\right\rangle$ $\in$ {$\left| {\alpha _V}\right\rangle$,$\left| {\alpha _H}\right\rangle$,$\left| {\alpha _D}\right\rangle$,$\left| {\alpha _A}\right\rangle$} and $\left| {\beta }\right\rangle$ $\in$ {$\left| {\beta _V}\right\rangle$,$\left| {\beta _H}\right\rangle$,$\left| {\beta _D}\right\rangle$,$\left| {\beta _A}\right\rangle$}. In the scheme, the signal state $\left| {\alpha }\right\rangle$ carries two-bit messages: $\left| {\alpha _V}\right\rangle$ = 00, $\left| {\alpha _H}\right\rangle$ = 01, $\left| {\alpha _D}\right\rangle$ = 10, and $\left| {\alpha _A}\right\rangle$ = 11. Alice simultaneously sends out the two orthogonal polarization states. At the sending side, the states are separated by the controllable polarization beam splitter (**cPBS**). The state $\left| {\beta }\right\rangle$, which is directed along the lower arm of the interferometer, undergoes a rotation of 90-deg. For instance, the rotation transforms the polarization of $\left| {\beta }\right\rangle$ from horizontal to vertical. At the receiving side, $\left| {\beta }\right\rangle$ is sent through a mainly transmitting beam splitter (**BS**) to detector **D1**. A small fraction of $\left| {\beta }\right\rangle$, equal to $\left| {\alpha }\right\rangle$, is reflected off (**BS**) and forwarded to interfere with $\left| {\alpha }\right\rangle$ at the upper **BS**. At the upper arm of the interferometer, the signal state $\left| {\alpha }\right\rangle$ undergoes two phase shifts (one by Alice and one by Bob) resulting in a state $\left| {e^{i\phi }\alpha }\right\rangle$, where $\phi$ indicates the difference between the phase shifts ($\phi$ = |**PSA** − **PSB**|). The phase shifts **PSA** and **PSB** could take two values (0-deg and 180-deg). These values are chosen at random. Note that Alice’s choice is independent of Bob’s choice. Based on $\phi$, the interference between reflected $\left| {\beta }\right\rangle$ and $\left| {e^{i\phi }\alpha }\right\rangle$ results in a click at either *X*-basis measurement system (**D2** or **D3**) or *Z*-basis measurement system (**D4** or **D5**). The latter is noted in Ref.^51^: **BS2** is used to discriminate $\left| {\alpha }\right\rangle$ and $\left| {-\alpha }\right\rangle$. In the context of Fig. 4, if the signal state is of the form $\left| {e^{i\pi }\alpha }\right\rangle$ = $\left| {-\alpha }\right\rangle$ ($\phi$ = 180-deg), a count occur in the *Z* measurement basis; in the other case ($\phi$ = 0-deg), a count occurs in the *X* measurement basis. In other words, this feature of the scheme is used to discriminate *Z* and *X* measurement bases. If there is no count at neither of the detectors, Bob considers the case as a measurement with an inconclusive result. In turn, **D1** is used as a reference, which indicates when a relevant measurement should be expected at the upper detectors. In other words, as mentioned in Ref.^51^, this detector is used as a trigger for the other two detectors. A requirement of this scheme is that an eavesdropper should send a $\left| {\beta }\right\rangle$ state even though she obtains an inconclusive result in her measurement. As noted in Ref.^51^, this will result in random counts in the detectors, i.e., the presence of the eavesdropper becomes detectable.

In order to perform the proposed relativistic protocol, Bob announces his phase shifts to Alice. Then, based on Bob’s phase shifts, her phase shifts, and bases (phase encoding) of the transferred WCPs, Alice determines via Eq. (2) which WCPs should be sifted (discarded) due to basis misalignment. This is the same procedure of sifting that was introduced in section “Key distribution scheme”. Next Alice publicly announces her sifting decisions and thus informs Bob about the WCPs having to be discarded. In this way, Alice and Bob obtain sifted key, which should be entirely correlated. Hereafter, parameter estimation, key reconciliation, and privacy amplification are perform by the two parties, as described in “Relativistic QKD protocol”.

We should note that the measurements conducted by Bob are time-sensitive, as in the original one-photon scheme of the proposed relativistic QKD: in order to meet relativistic conditions^42,43^, a WCP should be measured at a certain time $t_B''$ (see Fig. 1). Otherwise, a measurement is considered as inconclusive.

For the sake of clarity, an example, which shows the establishment of sifted key via the introduced WCP scheme, is presented. Suppose Alice generates the following two-bit symbols

$$\begin{aligned} 00\, 01 \,00\, 10\, 10\, 11. \end{aligned}$$

According to this sequence, Alice prepares the following WCPs

**Table:** 

$\left| {Q}\right\rangle _0$	$\left| {Q}\right\rangle _1$	$\left| {Q}\right\rangle _2$	$\left| {Q}\right\rangle _3$	$\left| {Q}\right\rangle _4$	$\left| {Q}\right\rangle _5$
$\left| {\alpha _V}\right\rangle$	$\left| {\alpha _H}\right\rangle$	$\left| {\alpha _V}\right\rangle$	$\left| {\alpha _D}\right\rangle$	$\left| {\alpha _D}\right\rangle$	$\left| {\alpha _A}\right\rangle$
$\left| {\beta _H}\right\rangle$	$\left| {\beta _V}\right\rangle$	$\left| {\beta _H}\right\rangle$	$\left| {\beta _A}\right\rangle$	$\left| {\beta _A}\right\rangle$	$\left| {\beta _D}\right\rangle$
0	0	0	1	1	1

where $\left| {Q}\right\rangle$ encompasses the pair ($\left| {\alpha }\right\rangle$,$\left| {\beta }\right\rangle$). The last row indicates the bases of the states (0 $\rightarrow$ *Z* basis; 1 $\rightarrow$ *X* basis). Alice sends to Bob these states via the interferometric scheme presented in Fig. 4. Alice chooses at random her phase shifts **PSA**$_i$:

**Table:** 

PSA$_0$	PSA$_1$	PSA$_2$	PSA$_3$	PSA$_4$	PSA$_5$
0-deg	180-deg	180-deg	0-deg	180-deg	0-deg

Suppose Bob at random chooses the following **PSB**$_i$ phase shifts:

**Table:** 

PSB$_0$	PSB$_1$	PSB$_2$	PSB$_3$	PSB$_4$	PSB$_5$
180-deg	0-deg	180-deg	0-deg	0-deg	0-deg

Based on the phase shifts of Alice and Bob, the $\left| {\alpha }\right\rangle$ states interfere with $\left| {\beta }\right\rangle$ states in a way that the following detectors click

**Table:** 

$\left| {Q}\right\rangle _0$	$\left| {Q}\right\rangle _1$	$\left| {Q}\right\rangle _2$	$\left| {Q}\right\rangle _3$	$\left| {Q}\right\rangle _4$	$\left| {Q}\right\rangle _5$
$D_4$	$D_5$	$D_2$ or $D_3$	$D_2$	$D_4$ or $D_5$	$D_3$

The case in which one or another detector is involved in the measurement process indicates that the measurement result is a probabilistic variable having uniform distribution. Note that in such cases, the recipient receives erroneous states (messages). Bob publicly announces his phase shifts **PSB**$_i$. Based on $\left| {Q}\right\rangle _i$, **PSA**$_i$, and **PSB**$_i$, Alice determines via Eq. (2) which WCPs should be sifted (discarded) due to basis misalignment. In the example, Alice informs Bob that $\left| {Q}\right\rangle _2$ and $\left| {Q}\right\rangle _4$ should be discarded (*reason*: uncorrelated bases are chosen by Alice and Bob). In this regards, the sifted key is formed by $\left| {Q}\right\rangle _0$, $\left| {Q}\right\rangle _1$, $\left| {Q}\right\rangle _3$, and $\left| {Q}\right\rangle _5$. It has the form

$$\begin{aligned} 00\, 01 \,10\, 11. \end{aligned}$$

The above example shows that it is possible to construct a WCP scheme, which is able to implement the proposed relativistic protocol. As shown above, the introduced interferometer enables a transfer of two-bit message (via one use of the scheme) in the case of quantum key distribution scenario. Also, it enables a sifting procedure, which is the same as that proposed in section “Key distribution scheme”. These features of the WCP scheme imply that this implementation could be assumed to be identical to the single-photon scheme described in Fig. 1.

## Summary

In summary, the paper reports an one-photon relativistic quantum key distribution protocol that is based on using an interferometric scheme (Mach–Zehnder interferometer) to transfer data (key bits) from one party to another. To be utilized in a key distribution system, the interferometer includes phase shift at each of its arms: one phase shift is controlled by the sender, whereas the other phase shift is controlled by the recipient. The proposed protocol takes as a basis the standard relativistic QKD protocols^42,43^. As shown above, the interferometric-based relativistic protocol is able to transfer two-bit messages via a single photon, i.e., its rate is twice the rate of the standard schemes, where a photon transfers just one bit. This results in obtaining greater size of the sifted key that is established during the protocol. This contribution follows from the usage of an one-photon interferometer, which is transformed into a *basis selection scheme* by introducing phase shifts at its arms and distinct basis measurement system at each output (*X*-basis measurement system at one output; *Z*-basis measurement system at the other output). Based on the phase shifts values, it is possible for correlated message transfer, which uses more than one basis, to be realized, as verified in the previous sections of this work. An analysis in terms of the security of the proposed protocol is put forward. It verifies the security against three attacks: intercept-resend attack, intercept-resend attack with preliminary prepared state, and collective attack. We quantitatively show that the novel relativistic protocol doubles the performance of its standard counterparts. The performance takes into account the rate of data transfer (in [*bits/qubit*]) and the size of the sifted key (in *bits*) established in the protocol. This conclusion is derived from comparing the characteristics of novel and standard protocols, as depicted in Table 1. A practical implementation of the proposed protocol is presented. The implementation is based on transferring weak coherent pulses via an interferometric scheme, which could discriminate two phase-encoding bases. This practical scheme is an adaptation of the scheme proposed in Ref.^51^; only slight changes are introduced in the way of applying phase shifts and sifting coherent states, which are uncorrelated between Alice and Bob.
